# Delayed skin reactions and mRNA vaccines against SARS-CoV-2: correspondence

**DOI:** 10.17179/excli2022-5481

**Published:** 2022-10-26

**Authors:** Rujittika Mungmunpuntipantip, Viroj Wiwanitkit

**Affiliations:** 1 Private Academic Consultant, Bangkok Thailand; 2 DY Patil Medical College, Pune, India

## ⁯⁯⁯

We would like to share ideas on the publication “Delayed skin reactions [DSR] after the second dose of mRNA vaccines against SARS-CoV-2 (Martins-Filho and Tenório, 2022[4])”. The data on the occurrence of DSR following the second dose of mRNA vaccines (BNT162b2 or mRNA-1273) against SARS-CoV-2 was assessed by Martins-Filho and Tenório (until June 17, 2022) (Martins-Filho and Tenório, 2022[4]). According to Martins-Filho and Tenório, about one-third of DLR instances may develop with the second dose of mRNA vaccinations, albeit this percentage may be understated due to possible reporting bias. Additionally, after the first dose, recurring reactions are not unusual and occur in roughly 25 % of individuals (Martins-Filho and Tenório, 2022[4]). Before making a conclusion on data from the case with clinical problem after vaccination, co-morbidity must be ruled out (Joob and Wiwanitkit, 2019[2]). For instance, thrombohemostatic disease is a clinical condition that may coexist in a recipient of the vaccination (Kebayoon and Wiwanitkit, 2021[3]). It is also impossible to totally rule out the possibility of asymptomatic SARS-CoV-2 that might be associated with skin lesions. Recent research has found a link between recipients' immunological reactions to vaccination and underlying genetic variation (Čiučiulkaitė et al., 2022[1]). The effects of genetic variation should be assessed if a further study is planned.

## Declaration

### Conflict of interest

None.

### Funding 

None.
